# Unpaid caregiving and stress among older working-age men and women in Sweden

**DOI:** 10.1016/j.ssmph.2023.101458

**Published:** 2023-06-22

**Authors:** Maria Stanfors, Josephine Jacobs

**Affiliations:** a Centre for Economic Demography, Lund University, P O Box 7080, 220 07, Lund, Sweden; b Health Economics Resource Center, Veterans Health Administration, Palo Alto, CA, USA

**Keywords:** Unpaid caregiving, Stress, Gender, Time use data, Linear probability models

## Abstract

Many individuals are experiencing the potentially stressful combination of providing care while still employed. In this study, the association between unpaid caregiving to another adult and self-reported stress among men and women aged 45–74 is investigated, using nationally representative time use diary data for Sweden (2000-01 and 2010–11, N = 6689). Multivariate regression analyses established that women were overall more stressed than men with the largest gender stress gap observed among intensive caregivers, providing >60 min of daily care and employed caregivers. The association between unpaid caregiving, employment, and self-reported stress is gendered. Among men, there is no caregiver effect regarding stress, but for women there is a net effect of 6–9%. Combining employment and unpaid caregiving (especially if intensive) is stressful for women but not for men. There are two potential mechanisms for this: less time for leisure and sleep. Unpaid caregiving is positively associated with stress among women when seen in relation to the way caregivers trade off time, not least to aid their recovery. These findings provide a more nuanced understanding of the time trade-offs carers make and uncover gender differences in the association between caregiving and stress that add to an existing gender stress gap. Given that unpaid caregivers are an important source of long-term care services, policymakers should consider that caregiving may be stressful and that stress impacts are gendered when designing and evaluating policies for longer working lives.

## Highlights

• Women in Sweden are more stressed than men, particularly high-intensity caregivers.
• Combining employment and unpaid caregiving is stressful for women but not for men.
• Women caregivers' stress is associated with leisure and sleep trade-offs.
• Caregivers' time trade-offs add to an existing gender stress gap.
• Respite care and coping assistance may help caregivers experiencing stress.

## 1 Introduction

Population ageing is increasing the demand for long-term care services, a trend which will continue as the population aged 80 and over increases in the coming decades. Unpaid caregivers are often the main source of long-term care services for ageing adults (Barczyk & Kredler, 2019; Freedman et al., 2023), providing an important service to recipients while offsetting significant costs to health care systems (Shahly et al., 2013; Uccheddu et al., 2019). Demographic and economic developments, such as increased longevity and women's increased labor force participation, raise questions concerning how unpaid caregivers will handle the competing demands of participating in the labor market while caring for the elderly, a combination which can be stressful for caregivers (Opree & Kalmijn, 2012).

The impact of unpaid caregiving on caregivers' health and well-being and the gender differences arising from this are contested topics (Sharma et al., 2016; Vlachantoni et al., 2016). Most empirical evidence suggests that unpaid caregiving, especially intensive caregiving, negatively impacts caregivers’ mental and physical health and that it affects women more than men (Bom et al., 2019b; Hiel et al., 2015; Hirst, 2005; MacDonald et al., 2005; Phillips et al., 2009; Pinquart & Sorensen, 2003; Uccheddu et al., 2019). Yet there are important gaps in the literature related to unpaid care and stress and how gender impacts this relationship.

The most common types of caregiver burden relate to psychological distress and devoted time (Viana et al., 2013), but few studies examine the intersection between the two. Previous research on caregiver stress often compares the stress of caregivers and non-caregivers, giving mixed results due to both methodological and conceptual challenges including small and biased samples, heterogeneity in the caregiving experience, or the over-representation of some high-risk caregivers (Kramer, 1997; Pinquart & Sorensen, 2003; Sharma et al., 2016). The latter can imply limited generalizability beyond caregivers in high-stress contexts (Kaschowitz & Brandt, 2017; Schulz et al., 1997). Such caregivers are often older or dedicated to spousal caregiving and are thus facing different time conflicts and trade-offs (with different implications for health) than working-age caregivers who primarily help their elderly parents (Bom et al., 2019a; Chappell et al., 2014). Research using nationally representative data to examine the relationship between caregiving and stress exists (Bom et al., 2019b; Hirst, 2005; Uccheddu et al., 2019), but it has not focused on the time use trade-offs that follow from caregiving responsibilities and that may relate to caregiver stress.

Existing research suggests that these time use trade-offs may be important considerations in understanding the relationship between unpaid care and stress. First, adapting one's time to accommodate unpaid care can be offset by reducing time in activities which are positively associated with mental and physical health, such as sleep and leisure. Increased levels of sleep disturbance have been associated with stress (Meltzer & Mindell, 2007), and unpaid caregivers have been found to experience increased sleep disturbances in cross-sectional studies of caregivers for Parkinson's sufferers (Happe & Berger, 2002) as well as in longitudinal research on employed caregivers in Sweden (Sacco et al., 2017). Caregivers in Sweden have also been shown to reduce leisure time (Stanfors et al., 2019), which is detrimental to physical and mental health (Jonsdottir et al., 2010) and is also indicative of a reduction in social and recreational activities – both important coping mechanisms and sources of recovery for caregivers (Pearlin et al., 1990). Despite the likely importance of these mechanisms in understanding caregiver stress, time use evidence of the relationships between sleep, leisure and stress has received little empirical attention in the literature on unpaid care given to other adults (MacDonald et al., 2005 being an exception). Second, caregiving can be stressful in ways concerning labor supply; a substitution away from paid employment can be stressful due to a reduction in household income and concerns about the ability to make ends meet (Pearlin et al., 1990). It can also be due to an increased loss of self (Skaff & Pearlin, 1992). Attempting to juggle employment and caregiving responsibilities tightens the time constraints of a 24-h day, which can induce stress through the sense of always being rushed (Hamermesh & Lee, 2007).

This study fills in the above noted research gaps by using nationally representative time use surveys from Sweden (2000-01 and 2010–11) and by analyzing a sub-sample of men and women aged 45–74 who filled out time diaries (N = 6689). The data contain a substantial number of caregivers, defined as individuals who provided unpaid care to another adult (within or outside the household) on the diary day (n = 1197). Our objective was to compare caregivers to non-caregivers, examining the relationship between self-reported stress as one important aspect of caregiver mental health and time allocation for activities associated with health and mental well-being, including leisure (Jonsdottir et al., 2010; Pearlin et al., 1990) and sleep (Meltzer & Mindell, 2007). We assess these relationships from a gender perspective in the Swedish policy context, which is known for extensive public support for work-care compatibility and a high degree of gender equality in the labor market and household. Specifically, we address the following research questions: 1) Is unpaid caregiving associated with self-reported stress, and does this association differ for men versus women? 2) To what extent is caregiving intensity associated with the likelihood of experiencing stress for men versus women? 3) Are there differential associations between stress and time allocation in paid work, routine housework, leisure, and sleep for caregivers versus non-caregivers, and do these associations differ by gender? Uncovering such mechanisms will further our understanding of caregiver stress and may inform policies to reduce caregiver stress.

## 2 Background

### 2.1 National context

Though men are increasingly providing unpaid care, women are more likely to take on caregiving roles throughout the life course than men, even if they are employed (Bertogg & Strauss, 2020). Women's increased labor force participation suggests the trade-offs between care provision and labor supply are intensifying. More than half of all caregivers must strike a balance between employment and care responsibilities (Sinha, 2013; Yeandle et al., 2006), and there is causal evidence that such a combination is stressful for middle-aged women (Opree & Kalmijn, 2012).

Sweden is no exception to these trends, as a large share of its population is aged 65+ (19.8% in 2015) (http://stats.oecd.org). The challenge of an ageing population is met by significant spending on policies supporting caregivers as a proportion of its GDP. This partly stems from the provision of needs-based cash allowances and services, which are available to both caregivers and care recipients. In the early 2000s, Sweden experienced a large shift towards in-home caregiving. The share of elderly in care facilities is decreasing, though the number of beds in nursing and care facilities, per capita population aged 65+, is higher in Sweden than in, for example, the UK. The increasing demand for unpaid care in Sweden is occurring in a context where policy makers have aimed to promote gender neutral policies and the dual-earner/dual-carer model. Sweden was an early adopter of this model, which aims to ease work-family conflicts through universal leave programs, income support, and publicly funded care facilities and services. This combination of health and labor market policies provides a unique context for assessing the relationship between time use and stress for unpaid caregivers.

### 2.2 Theoretical considerations

This research is guided by time allocation theory, which posits that individuals with family and household responsibilities must trade off among three time uses: paid work, leisure, and home care (Becker, 1965; Gronau, 1977). Time allocation models consider decisions on labor supply and care as interrelated because they compete for the caregiver's time, which is limited to 24 h per day. Individuals are assumed to rationally choose the optimal amount of time for different activities and the resources they need to maximize their utility. The model predicts that individuals allocate their time so that an extra hour (on the margin) renders the same utility irrespective of whether it is spent in paid work, leisure, or unpaid (e.g., care) work. All else equal, an increase in the marginal utility of paid work leads to a reduction of leisure time and unpaid work, and vice versa. Gender differences and gendered impacts of unpaid work, including caregiving, are predicted to be in line with specialization based on relative productivity differences. This is consistent with labor market segregation, where women are more likely to be employed in jobs with characteristics that enable them to take on a caregiving role (Carmichael & Charles, 1998; Jacobs et al., 2017).

Preferences may also play a role. That carers may prefer more leisure or even more paid work to escape caregiving responsibilities is known as a “respite” effect (Carmichael & Charles, 1998; Heitmuller, 2007). If unpaid caregiving and leisure are complements, caregiving may increase the value of leisure and render small and even positive effects. From a time allocation perspective, stress may arise from the tensions created in that the individual cannot attain the optimal balance of activities or in that there are inadequate hours in the day to perform the tasks at hand (Hamermesh & Lee, 2007). Caregiving is one activity where the individual may lack autonomy over the timing and the time required to care for others (Kaschowitz & Brandt, 2017; Uccheddu et al., 2019), which intensifies the trade-offs faced. Higher caregiving burden and lack of control have been associated with higher levels of stress, especially in contexts with less public support for carers and a low supply of respite care and long-term care options (Wagner & Brandt, 2018). The tension arising from time pressure is consistent with sociological theories on role strain, role overload and conflict, which would provide similar predictions (Pearlin et al., 1990; Penning & Wu, 2016; Skaff & Pearlin, 1992).

Based on these theoretical considerations, we expect caregivers, especially intensive caregivers, to be more stressed than non-caregivers. Because women generally take on more caregiving responsibilities across the life course, we expect women caregivers to be more stressed than men. While we expect stress to be associated with caregivers’ everyday time allocation, we do not expect time in paid work to be associated with caregivers experiencing stress because of the high degree of work-care compatibility in Sweden. We expect negative associations between time in leisure and sleep and self-reported stress to be of particular importance for caregivers.

## 3 Methods

### 3.1 Data

Data come from the Swedish Time Use Survey (SWETUS, maintained by Statistics Sweden) 2000-01 and 2010–11, which are the most recent surveys ready for analysis. The sample is nationally representative and includes weights to control for over- and under-sampling of certain population groups. SWETUS was conducted at the individual level. The sample was restricted to individuals aged 45–74, who filled out one or two diaries of 1440 min covering at least five daily activities.^1^ The self-completed time diary is considered the most reliable and accurate way to collect data on the activity patterns of large populations based on probability samples. A key strength of the methodology is that it does not suffer from recall bias but there are other advantages (see Michelson, 2005 and Robinson & Godbey, 1999 for more detailed discussions). Ages 45–74 were selected because the focus is on older working-age men and women and those who could potentially be working by postponing retirement. Members of this age group are typically not ailing from the age-related conditions that occur later in life. Though most men and women in this age category did not have young children at home to care for, many faced caregiving responsibilities towards other adults, which could interfere with work and other activities. Including younger individuals with childcare responsibilities would have confounded the relationship between stress and unpaid caregiving, especially from a gender perspective. Our total sample size is 6689 individuals of which 1197 were caregivers.^2^

### 3.2 Analytic strategy

The analyses focus on how unpaid caregiving is associated with stress and the extent to which gender, alone or in combination with caregiver status or caregiving intensity, determines stress experiences in Sweden. To assess whether caregivers experienced more stress than non-caregivers and potential gender differences in these associations, we determined the (weighted) proportion of men and women experiencing stress by caregiver status. Then, we applied linear probability models (LPM) to study the probability of stress with caregiving and gender as the independent variables of interest.^3^ Gender and caregiver status were interacted to determine whether the association between caregiving and stress differed significantly by gender. Models controlled for gender, a vector of controls including sociodemographic factors (**X**_i_), diary day, and survey year:

$${Stress}_{i}=\alpha +\beta_{1}{Caregiver}_{i}+\beta_{2}{Gender}_{i}+\beta_{3}Caregiver\times {Gender}_{i}+\beta_{4}X_{i}+\beta_{5}Diary\;{day}_{i}+\beta_{6}{Year}_{i}+\varepsilon_{i}$$

Additional analyses restricted our sample to employed individuals because they are more likely to suffer from work-related stress as well as from caregiver stress and role overload. We then replicated these analyses substituting intensive caregiver status (i.e., providing more than 60 min of care a day) with caregiver status. Finally, because caregiving may be associated with trade-offs that make (intensive) caregivers time-poor through overload with less time for recovery (e.g., through reduced leisure or sleep), we exploited the advantage of time use data to better understand the association between stress experience and time allocation. As men and women still have quite different time use patterns, especially when middle-aged and older, with women doing less paid work but more unpaid work, the analysis of stress and caregiving and the interaction between the two was stratified by gender. We used ordinary least squares regressions to assess whether there were differential associations between stress and time allocation in paid work, routine housework, leisure, and sleep for caregivers versus non-caregivers.^4^ In these regressions, the number of minutes spent for each activity was the dependent variable, and caregiving status was interacted with an indicator for stress experience.

### 3.3 Variables

*Self-reported stress* is the primary outcome variable for the analyses addressing the first two research questions*.* A binary variable was created to indicate individuals who answered yes to the diaryday specific question: *“Have you felt stressed during the diary day?”*. This is a good measure identifying the relationship between caregiving and stress because it asks the individual about feeling stressed on the very day that the unpaid caregiving (and related time allocations) took place. The stress question has minimal non-response (1.6%). Diaries which did not include responses to the stress question were dropped from the sample.

Our main independent variables are gender and caregiver status and intensity. These variables were interacted to test whether the association between caregiving and stress differed significantly by gender. Using the time diaries, we identified unpaid *caregivers* based on the individual having provided 10 min or more of caregiving to another adult either within or outside the household on the diary day (we are unable to identify the caregiving recipient, despite its relevance). This included activities such as physical care, help with meals, accompanying the adult to hospital, emotional care, and transport (see Appendix Table A1 for variable coding). Transport was included because it is the most common caregiving task (Sinha, 2013). Defining caregivers exclusive of transport would reduce the number of caregivers considerably but would also better capture personal caregiving, which is more demanding both mentally and physically, and may impact caregivers’ health more negatively than other types of caregiving (Hiel et al., 2015). High-intensity caregivers are defined as those who provided at least 60 min of caregiving daily.

For analyses exploring time costs and trade-offs with other daily activities (i.e., the third research question), all outcomes are expressed in daily minutes. *Paid work* is considered work for pay outside or within the home. Commuting time was excluded to avoid overstating time for part-time workers. *Routine housework* includes tasks such as cooking/washing up, cleaning, laundry, shopping, and domestic travel (for performing these duties). *Leisure* includes passive and active leisure as well as socializing. It includes indoor and outdoor sporting and leisure activities (e.g., walking, cycling, exercise), outings such as going to the cinema, sporting events, the theatre, or other public events, social meetings with friends, artistic or musical activities, crafts and hobbies, restaurant visits, and television viewing. *Sleep* includes night-time sleep but not naps.

Control variables were theoretically motivated individual and household characteristics that have been shown to be associated with self-reported stress, including age, education and employment status, household type, children under 19 in the household, and partner's employment status. As endogeneity issues relating to caregiving and time allocation decisions can be problematic, income is not included in the analysis.

## 4 Results

Table 1 presents a descriptive overview of all the individuals in the sample and of the individuals providing unpaid care for another adult during the diary day. We identified 1197 diaries from individuals who provided unpaid care on the diary day, which equates to 17.9% of the sample. Of note, the observable characteristics of caregivers were like those of the overall sample. It should also be noted that unpaid caregiving to another adult is gender-equal in Sweden (more so than elsewhere, see Stanfors et al., 2019). Most caregivers were low-intensity caregivers. Caregivers defined as providing intensive caregiving (i.e., more than 60 min during the diary day) comprised 33% of all caregivers. 13% of the sample reported experiencing stress on the diary day, but, on average, caregivers were not more likely to be stressed, based on descriptive statistics.

**Table 1:** Sample characteristics, weighted means and proportions for full sample and caregivers only.

	Full sample	Caregivers
Sex		
Man (ref.)	0.48	0.50
Woman	0.52	0.50
Caregiver (>0 min/day)		
No (ref.)	0.82	0.00
Yes	0.18	1.00
Intensive caregiver (>60 min/day)		
No (ref.)	0.94	0.67
Yes	0.06	0.33
Stress		
No (ref.)	0.87	0.87
Yes	0.13	0.13
Age		
45-54 (ref.)	0.38	0.36
55–64	0.36	0.37
65–74	0.26	0.27
Education		
Primary only (ref.)	0.23	0.23
Secondary	0.44	0.44
Higher	0.33	0.33
Employment status		
Full-time work (ref.)	0.52	0.50
Part-time work	0.13	0.14
Other/not in paid work/retired	0.35	0.37
Household type		
Single (ref.)	0.22	0.19
Partnered	0.78	0.81
Child under 19 in household		
No (ref.)	0.79	0.81
Yes	0.21	0.19
Employment status partner		
Employed (ref.)	0.42	0.45
Retired	0.31	0.32
Other	0.08	0.08
No partner	0.18	0.15
Household income		
Lowest 25% (ref.)	0.19	0.17
Middle 50%	0.51	0.52
Highest 25%	0.30	0.31
Year		
2000–01 (ref.)	0.50	0.54
2010–11	0.50	0.46
N	6689	1197

*Notes:* Figures subject to rounding. See Appendix Table A1 for details on coding of stress and caregiving variables.

### 4.1 Are caregivers more stressed than non-caregivers?

Table 2 presents more detailed information regarding the prevalence of self-reported stress among non-caregivers and caregivers by gender. Among men, non-caregivers were more likely to report stress than those who were caregivers. Among women, those who provided care during the diary day were more likely to report stress. Women were significantly more likely to report stress during the diary day than were men, irrespective of caregiver status. When looking only at employed individuals, women were significantly more likely to report stress than men, especially if they were caregivers. The prevalence of stress was highest among employed women who were also caregivers, thus gender differences were larger among employed caregivers.

**Table 2:** Self-reported stress among men and women aged 45–74 (non-caregivers and caregivers) for full sample and those employed only.

Full sample	Non-caregivers	Caregivers
Men	10.4	8.6
Women	14.3***	17.1***
N	5492	1197

Employed	Non-caregivers	Caregivers
Men	11.1	8.8
Women	13.0*	18.9***
N	3926	754

*Notes:* Prevalence of self-reported stress is weighted. Statistically significant gender differences are indicated: *p < .10, **p < .05, ***p < .01.

Linear probability models assessing whether caregivers were more likely to experience stress than non-caregivers are summarized in Table 3. Models were estimated for the full sample and for the employed only. Panel A summarizes the results for models including an interaction term (*caregiver* × *gender*) to test for gender differences in the caregiver effect on stress for all caregivers, while Panel B summarizes models with an interaction (*intensive caregiver* × *gender*) for those providing care more than 60 min per diary day. The coefficients presented in Table 3 show the impact of one variable when the other variable being part of the interaction is zero. The base effect of caregiving (intensity) indicates the association for men (reference category for gender). The woman coefficient shows the difference in stress probability between women and men who do not provide (intensive) unpaid caregiving. The interaction effect shows the additional caregiving (intensity) effect, if any, for women. To get the net effect of caregiving (intensity) for women, the base and interaction effects must be added.

**Table 3:** Results from linear probability models demonstrating the extent to which caregivers are more stressed than non-caregivers among men and women aged 45–74.

A Caregivers (any amount of care) versus non-caregivers		
	Full sample	Employed only
Gender (Man ref.)		
Woman	0.041*** (0.010)	0.019 (0.012)
Caregiver (No ref.)		
Yes	−0.015 (0.014)	−0.020 (0.017)
Interaction Yes × woman	0.074*** (0.018)	0.088*** (0.023)
N	6689	4680
B Intensive caregivers (>60 min per day) versus non-caregivers		
	Full sample	Employed only
Gender (Man ref.)		
Woman	0.041***(0.010)	0.019(0.012)
Intensive caregiver (No ref.)		
Yes	−0.011 (0.024)	−0.013(0.031)
Interaction Yes × woman	0.067** (0.029)	0.104***(0.039)
N	6689	4680

*Notes*: Estimates from models controlling for all variables listed in Table 1 except household income. Panel B models also consider non-intensive caregiving (see Appendix Table A2).

*p < .10, **p < .05, ***p < .01.

Estimates shown in Table 3 confirm that among those who did not provide care to another adult, women were more likely than men to report stress, net of observables. This is, however, sensitive to employment, with no gender difference in terms of self-reported stress among the employed, indicating that the gender difference in Table 2 is explained by observable characteristics. There is no statistically significant difference in stress probability between men who were caregivers and comparable men who were not caregivers. This finding holds for any caregiving as well as for caregiving intensity (similar zero estimates) and is unrelated to employment. The interactions between being a caregiver and a woman are, however, both statistically significant and large, which means that women who provided unpaid caregiving to another adult during the diary day were considerably more likely to report stress than other comparable women. Net effects indicate that women who provided unpaid caregiving to another adult were about 6–9% more likely to report stress than women who did not provide such care. This holds for any unpaid caregiving or intensive caregiving, and irrespective of whether the full sample or the employed only are analyzed (β = −0.015 + 0.074 and β = −0.020 + 0.088 if any caregiving and β = −0.011 + 0.067 and β = −0.013 + 0.104 if intensive caregiving). Net effects were largest when caregiving intensity was considered among the employed (9.1%).^5^ These results resonate with previous findings showing that, in Sweden, most individuals with caregiving responsibilities stay in the labor force and try to combine work and caregiving through flexible work schemes and through taking recourse to public support for long-term care (Stanfors et al., 2019). Obviously, this associates with more stress experiences among women.

If we are interested in how the probability to report stress during the diary day among women who provided care to another adult compares to that of men who were caregivers, we need to add the coefficients for ‘Woman’ and the interaction term and multiply with 1. Results from this exercise show that unpaid caregiving adds to gender differences in stress. Women were 10.7–12.3% more likely to report stress associated with caregiving than men (β=(0.041 + 0.074)x1 and β=(0.019 + 0.088)x1 for any caregiving and β=(0.041 + 0.067)x1 and β=(0.019 + 0.104)x1 for intensive caregiving). The gender difference in stress was largest among the employed who were intensive caregivers (12.3%). These estimates should be compared to the limited gender difference among those who did not provide care (4.1% for all, and basically zero for the employed).

### 4.2 How is stress associated with caregivers’ time allocation?

Table 4 shows that self-reported stress is positively associated with paid work for men – those who reported stress on the diary day worked on average 47 min more per day compared to comparable men. For women, stress is positively associated with time in routine housework – those who reported stress did 11 min more than comparable women. For both men and women, less leisure time was associated with being more likely to report stress during the diary day. For men, those who slept less were more likely to report stress. Of note, the associations between caregiver responsibilities and time use are similar for men and women in Sweden, though coefficient magnitudes differ. Caregivers devote less time to paid work and have less time for leisure but perform more routine housework, perhaps as part of the caregiving by servicing another adult with home-produced goods and services. It is evident from the interaction effect of unpaid caregiving and stress that there is a positive association with routine housework and a negative association with leisure and sleep (though the latter is only significant for women, yet suggestive for men). Again, coefficient magnitudes differ, and they should be interpreted against the backdrop of men's and women's average time use patterns. Women generally perform more routine housework than men and have less time for leisure. They may therefore react to the stressful addition of caregiver responsibilities by feeling more stressed from relatively smaller increases in housework or reductions in leisure time. Of note, there is no significant interaction effect of unpaid caregiving and stress versus paid work for men and women in Sweden.

**Table 4:** Results from ordinary least squares models demonstrating how stress is associated with caregivers’ time allocation and trade-offs among men and women aged 45–74.

	Men				Women			
	Paid work	Routine housework	Leisure	Sleep	Paid work	Routine housework	Leisure	Sleep
Stressed (Not stressed ref.)	46.6*** (14.0)	−2.0 (6.3)	−27.0** (12.6)	−12.6* (7.5)	15.4 (9.5)	11.1* (6.2)	−34.7*** (10.3)	−5.4 (5.6)
Caregiver (No ref.)	−46.8*** (8.3)	38.1*** (5.0)	−29.8*** (8.6)	−1.7 (4.7)	−38.1*** (7.4)	13.7*** (4.8)	−23.4*** (8.2)	−1.1 (4.7)
Interaction Caregiver × stressed	1.8 (23.6)	59.3*** (17.0)	−106.1*** (19.3)	−22.9 (14.1)	−20.6 (16.9)	30.9*** (9.8)	−28.2* (15.8)	−25.0** (9.7)
N	3082				3607			

*Notes:* See Table 3. Paid work regressions also excluded the employment status variable. *p < .10, **p < .05, ***p < .01.

## 5 Discussion

This study contributes to the literature on the relationship between unpaid caregiving for another adult and stress. It should be seen against the backdrop of demographic changes fueling uncertainty about the future supply of unpaid caregiving (Freedman et al., 2023), alongside population ageing increasing the need for labor supply across Europe and North America. To address these concerns, older adults, not least women, are an important and untapped resource, though it is unclear how unpaid caregiving needs will impact their labor supply. It is also unclear how the combination of paid work and unpaid caregiving associates with stress – a state which may affect older adults in the labor force.

It is thus vital to establish the links between gender, caregiving, and stress and to identify mechanisms at work. It is also important to establish what environments support those who provide unpaid caregiving to another adult. For these reasons we used nationally representative data for Sweden and compared middle-aged and older men and women (aged 45–74), employing time use surveys from the first decades of the 2000s. We asked whether caregivers are more stressed than non-caregivers, to what extent caregiving intensity is associated with self-reported stress and whether gender differences exist regarding these associations. We also examined the extent to which stress is associated with caregivers’ everyday time allocation. Even though the two most common types of caregiver burden are devoted time that crowds out other daily activities and psychological distress (including stress) (Viana et al., 2013), there are very few studies which look at the trade-offs that caregivers experience in relation to stress. This study fills that research gap. Moreover, in Sweden, stress-related symptoms are the primary reasons for sickness absence among women, with stress-related mental disorders, depression, and anxiety, accounting for 44% of long sick leaves in Sweden in 2017 (Svenningsson et al., 2022), and thus the topic of this study is highly policy-relevant.

To summarize, results established that, in Sweden, women are more likely to report stress than men, irrespective of caregiver status. While gender differences in stress are limited among non-caregivers, they are large among caregivers, especially among the employed who provide intensive caregiving to another adult. This is in line with expectations based on time allocation and stress process theories. A detailed examination of how gender shapes the association between unpaid caregiving (intensity) and self-reported stress shows that: Men who provided unpaid care to another adult during the diary day were not more likely to report stress than other comparable men who did not provide care. Women who provided unpaid caregiving were, however, considerably more likely to report stress than other comparable women. This result was stable for all and for those who were employed. The caregiver effect was particularly strong for employed women who provided intensive (>60 min per day) caregiving. The results show that women in Sweden who typically stay in the labor force while taking on caregiving responsibilities for another adult experience stress. This is in line with hypotheses relating to women's higher caregiving load and stress across the life course, and it is an example of competing time demands and role overload that become particularly acute for those who combine paid work with the provision of unpaid caregiving. In other contexts where women's lives are more structured around caregiving responsibilities, we would expect that those who provide (intensive) caregiving responsibilities for another adult typically leave the labor force and even co-reside with the care recipient. This may also be stressful but in other ways. Women may experience caregiving as more stressful, even when the context is oriented at gender equality and exposure to stressors is similar. Together, the different associations between unpaid caregiving, employment, and self-reported stress among Swedish men and women contribute to gender gaps in caregiver well-being in a more gender equal context than those previously explored (MacDonald et al., 2005; Uccheddu et al., 2019).

There are other factors, unmeasured in this study, that may explain this gender gap. In addition to women doing more unpaid work than men and having less time for recovery, it is also possible that otherwise similar intensity male and female caregivers differ regarding stress in part because they engage in different types of tasks when providing unpaid care. The fact that female intensive caregivers are more stressed could relate to their higher propensity to be conducting more stressful types of tasks (i.e., personal care) than men (Sharma et al., 2016) or due to a higher propensity for women to engage in multiple tasks simultaneously, which can result in increased stress (Zaiceva, 2022). It may also be that women caregivers receive less support either from a paid source or from a broader network of caregivers than men. Prior research has found that women are less likely than men to ask for support either from other sources of paid and unpaid care and that they are more likely to be providing care across multiple domains relative to men (Vicente et al., 2022; Zygouri et al., 2021).

Time use analyses revealed that, as expected, stress is associated with caregivers’ everyday time allocation. We found that unpaid caregiving and self-reported stress are associated with similar time use trade-offs for men and women in Sweden. Caregiver stress is associated with more routine housework irrespective of gender. Men and women caregivers who are stressed also suffer from less leisure (primarily men) and sleep (women) compared to other men and women who do not provide unpaid care to another adult. As hypothesized, the association (interaction) between caregiver stress and paid work is insignificant, which indicates work-care compatibility and that carers in Sweden do not face a trade-off that potentially makes them both time-poor and poor in terms of income. Nevertheless, and as hypothesized, caregivers trade off time in leisure activities and sleep that support individual well-being and recovery. The negative interaction effects between providing unpaid care to another adult and self-reported stress has implications for leisure and sleep, in a gendered way. These findings suggest that the positive association between unpaid caregiving and self-reported stress is related to the way caregivers trade off time for recovery through leisure and sleep. Such a trade-off can be an example of a mechanism which influences stress, as leisure is associated with improved mental and physical health (Jonsdottir et al., 2010). Also, sleep is a mechanism through which stress can be mediated, which is of importance not least for caregivers who also are employed (Happe & Berger, 2002; Sacco et al., 2017). The findings are important for both understanding gender differences in terms of stress and being aware of the need for respite care and active caregiver policies to avoid more severe caregiver stress, burnout, and depression. This study provides a more nuanced understanding of the types of time trade-offs individuals with caregiving responsibilities make, providing evidence that gender still structures caregiving across the life course with implications for individual well-being.

It is important to consider study limitations when interpreting these findings. Potential endogeneity between caregiver status and time spent in paid work and other activities should be acknowledged. Specifically, we note the possibility of reverse causality. For example, it is possible individuals who experience greater stress, perhaps due to work commitments, are more inclined to leave the labor force and become a caregiver. For paid work, studies have found that an assumption of exogeneity underestimates the effect of unpaid care on labor supply, while others find the opposite. Where there is an overestimation of this effect, it has been found to be minimal for higher intensity caregivers (Heitmueller, 2007), implying that this is more of a concern in analyses focused on the binary caregiving status variable. Some studies have found that fixed effects models sufficiently account for bias due to unobserved factors that do not change over time, which is another potential source of bias when examining the relationship between caregiving and labor supply (Van Houtven, Coe & Skira, 2013). As we did not have access to longitudinal data, we were unable to apply methods that accounted for unobserved time-invariant heterogeneity. Similar assessments have not been done for other time use outcomes due to lack of longitudinal data and valid instrumental variables. As such, this study makes no attempts to infer causality from the regression results.

Another limitation is that the data did not differentiate between caregiving activities and did not distinguish between help with grocery shopping and transport – tasks that are relatively easy both to perform and to fit into a daily schedule – and heavier caregiving involving personal care and providing end of life care, which has been shown to be more detrimental than other types of care concerning self-assessed health and the financial penalty incurred (Williams et al., 2014). In relation to this, transitions into care cannot be identified, despite them being stressful (Uccheddu et al., 2019). Moreover, the fact that the data build on reports for specific diary days means that we have underestimated the number of caregivers because not everyone who has caregiver responsibilities provides unpaid care every day. We acknowledge this shortcoming and the fact that we have some caregivers in our non-caregiver reference group which reduces statistical inference but, if anything, underestimates caregiver effects. We also note potential limitations of our measure of stress. First, while this measure captures self-reported stress on the diary day, stress on the day of data collection may be different from day-to-day time stress. Findings from Urwin et al. (2023) suggest that while evaluative well-being may be lower amongst caregivers, this differs significantly from daily average experienced well-being. Second, we note that this measure is a binary indicator of stress. Optimally, we would have used a measure with greater specificity which allowed for multiple dimensions of stress to be captured, as stress measures with multidimensionality tend to have stronger psychometric properties. Future analyses focused on stress and caregiver time use would benefit from using validated stress scales, such as the Perceived Stress Scale (Taylor, 2015), which would also allow for a more nuanced understanding of the type of stressors driving our results. Finally, we note that we were not able to control for self-reported health measures which may differ between caregivers and non-caregivers (Vitaliano et al., 2003).

## 6 Conclusion

The main conclusions from the present study are: Women are more likely to report stress than men in Sweden. Unpaid caregiving for another adult is associated with increased stress for women and creates important gender gaps in caregiver well-being, not least among the employed who are challenged with more than 60 min caregiving during the day. The findings were from a policy context that supports work-care compatibility and gender equality more than elsewhere, and where the Swedish government funds in-home care to a larger extent than other OECD countries. Nevertheless, in Sweden, unpaid caregiving is associated with stress, especially among women, though results likely indicate attenuated effects both regarding gender and caregiver status (including intensity) because of the supportive aspects of the Swedish welfare state compared to other contexts. Caregivers who also experience high levels of stress spend less time on leisure and sleep than do non-caregivers and caregivers who are not stressed. The results from this study illustrate that unpaid caregiving is associated with gendered health consequences in terms of stress, which policy makers should consider and seek to remedy at an early stage before stress experiences translate into caregiver burnout and depression. Given population ageing and the need to increase labor supply in a sustainable way, this is an increasingly important policy issue across Europe and North America.

## Ethical statement

Ethics approval from the regional ethics committee (EPN) was obtained for the present research (Dnr 2017 349).

## Author statement

Maria Stanfors: Conceptualization, Methodology, Data curation, Writing- Original draft preparation, Reviewing and Editing. Josephine Jacobs: Writing- Original draft preparation, Reviewing and Editing.

## Declaration of competing interest

None of the authors declare any conflicts of interest.

## Data Availability

The authors do not have permission to share data.

## Footnotes

1 We only use diaries which include at least five activities because this is considered a minimum for a good diary.

2 See Appendix Figure A1 for a description of how sample was obtained.

3 LPM estimates are more straightforward to interpret than those from logit models, especially interactions. It is also more straightforward to compare LPM estimates across sub-samples (e.g., by gender) than is the case for odds ratios from logit models.

4 This is one of the advantages of time use data, especially when collected through the diary method which is superior to all methods and considered “gold standard” in time use research. We thus cover the full day and not only one or two activities in the analyses.

5 Of note, in this comparison, results for the category providing non-intensive caregiving align well with those comparing caregivers providing any care to non-caregivers (see Appendix Table A2 for details).
