# Supplementary material for: Unpaid caregiving and stress among older working-age men and women in Sweden
Source: SSM Popul Health. 2023 Jun 22;23:101458. doi: 10.1016/j.ssmph.2023.101458 (PMC10310475; doi:10.1016/j.ssmph.2023.101458)
Supplement: Multimedia component 1 [file mmc1.docx]

APPENDIX

Figure A1. Sample flow diagram.

| Full sample:  14,432 diaries  (6,478 men and 7,954 women aged 15-88) |  |
| --- | --- |
|  | 7,562 diaries were excluded because they were younger than 45 or 75 and older |
| 6,870  (3,176 men and 3,694 women aged 45-74) |  |
|  | 41 diaries were excluded due to missing work status |
| 6,829  (3,165 men and 3,664 women aged 45-74) |  |
|  | 31 diaries were excluded because their level of education was unknown |
| 6,798  (3,145 men and 3,653 women aged 45-74) |  |
|  | 109 diaries were excluded because information on stress experience during the diary day was missing |
| Final sample: 6,689  (3,082 men and 3,607 women aged 45-74) |  |

*Source:* Swedish Time Use Survey 2000-01 and 2010-11.

Table A1. Variable coding (SWETUS 2000-02 and 2020-11).

| Coding stress variable | | |
| --- | --- | --- |
| Survey question | Original coding | Dependent variable |
| Have you felt stressed on the diary day?  (Har du känt dig stressad under dagboksdagen?) | Binary: No/Yes. | 1 if yes |
|  |  |  |
| Coding caregiver variable |  |  |
| Time use activities | Variable codes | Notes |
| 2410 Help for adults in own household 2421 Assistant to others’ households 2430 Hospital visit (related to others)  2499 Unspecified care for others  2040 Travel in connection with adults in own or another household | Activity variable codes 2410, 2421, 2430, 2499, 2040. | 2430 is hospital visit but we do not know who ‘ego’ is visiting.  2040 is travel in connection with assistance to or care for adults in own or another household but we do not know the reason. |

Table A2. Results from linear probability models demonstrating the extent to which caregivers are more stressed than non-caregivers among men and women aged 45-74.

| A Caregivers (any amount of care) versus non-caregivers | | |
| --- | --- | --- |
|  | Full sample | Employed only |
| Gender (Man ref.) |  |  |
| Woman | 0.041***  (0.010) | 0.019  (0.012) |
| Caregiver (No ref.) |  |  |
| Yes | -0.015  (0.014) | -0.020  (0.017) |
| Interaction Yes×woman | 0.074***  (0.018) | 0.088*** (0.023) |
| N | 6,689 | 4,680 |
| B Intensive caregivers (>60 minutes per day) versus non-caregivers | | |
|  | Full sample | Employed only |
| Gender (Man ref.) |  |  |
| Woman | 0.041***  (0.010) | 0.019 (0.012) |
| Caregiver (No ref.) |  |  |
| Non-intensive | -0.017 (0.017) | -0.023 (0.020) |
| Intensive | -0.011 (0.024) | -0.013 (0.031) |
| Interaction Non-intensive×woman | 0.078***  (0.021) | 0.081***  (0.027) |
| Interaction Intensive×woman | 0.067**  (0.029) | 0.104***  (0.039) |
| N | 6,689 | 4,680 |

*Notes*: Estimates from models controlling for all variables listed in Table 1 except household income.

*p < .10, **p < .05, ***p < .01.
*Source:* See Table 1.
